# Quality and dissemination of nasopharyngeal cancer health information on TikTok and Bilibili: Cross-sectional study

**DOI:** 10.1097/MD.0000000000048723

**Published:** 2026-05-08

**Authors:** Yang Miao, Hui Wu, Qineng Gong, Linlin Zhang, Tingting Shen, Ye Hu

**Affiliations:** a Department of Pharmacology, The First People’s Hospital of Yancheng, Yancheng, China; b Medical Research Center, Affiliated Hospital 2 of Nantong University, Nantong, China; c Department of Rehabilitation Medicine, Yancheng Third People’s Hospital, Yancheng, China.

**Keywords:** global quality score, modified DISCERN, nasopharyngeal cancer, quality assessment, short videos

## Abstract

Nasopharyngeal cancer is a cancer originating from the nasopharyngeal epithelium. Health education serves as an effective measure for prevention and treatment. With the rapid development of short videos, platforms such as TikTok and Bilibili have become primary sources for health information. However, the quality of nasopharyngeal cancer-related videos on these platforms remains unexplored. This study aims to evaluate the information quality of short videos related to nasopharyngeal cancer on the TikTok and Bilibili platforms. A cross-sectional study was conducted on October 1, 2025; the top 100 short videos related to nasopharyngeal cancer were collected from TikTok and Bilibili through searches in Chinese. After extracting basic information, the global quality score (GQS) and the modified DISCERN (mDISCERN) tool were used to assess each video’s quality and reliability. The GQS rating scale ranges from 1 (poor quality) to 5 (high-quality). The modified DISCERN scores range from 0 to 5, with higher scores indicating greater reliability. Additionally, Spearman correlation analysis was applied to examine relationships between video characteristics, GQS, and DISCERN scores. A total of 200 videos were included in the analysis, with 58.5% of the videos uploaded by healthcare professionals, 19.5% by science communicators, 14.0% by general users, and 8.0% by organizational users. The median GQS and mDISCERN scores for nasopharyngeal carcinoma videos were 3 and 2, respectively. TikTok videos showed significantly higher engagement compared to Bilibili, with median likes of 1018 vs 16, collections of 313 vs 14, comments of 206 vs 2, and shares of 282 vs 5 (all *P* < .001). No statistically significant differences were observed between TikTok and Bilibili in GQS or mDISCERN scores. The main content themes on both platforms were clinical manifestations and treatment, while prognosis was rarely discussed. Correlation analysis revealed strong correlations among interactive data points, but weak or negative correlations between interactive data and GQS or mDISCERN scores. The informational content and quality of nasopharyngeal cancer-related videos on TikTok and Bilibili require improvement. Enhanced management of health science popularization videos on short video platforms is needed to ensure the dissemination of accurate and reliable health information.

## 1. Introduction

Nasopharyngeal cancer is a malignant tumor originating from the mucosal epithelium of the nasopharynx, with obvious geographical and population distribution characteristics.^[1,2]^ In 2020, approximately 133,000 new cases and 80,000 deaths were reported globally, with over 75% occurring in Southeast Asia and southern China. The incidence rate among males was 3 times that of females.^[3]^ Due to the hidden location of the nasopharynx and the lack of obvious early symptoms, about 80% of patients are in advanced stages at the time of diagnosis.^[4]^ Therefore, early recognition, intervention, and treatment are particularly important to improve patient prognosis and increase the cure rate.^[5]^

Short videos present complex health issues in an easily understandable format, meeting the public’s demand for convenience and immediacy in understanding disease-related content.^[6]^ Studies have shown that active use of social media for disease-related information is associated with improved patient prognosis.^[7,8]^ Since its launch in September 2016, TikTok has rapidly become one of the world’s fastest-growing social media platforms. Bilibili went live in June 2009 and boasts a broad user base spanning all age groups, with content covering diverse topics including health, nutrition, and beauty.^[9]^ In recent years, short video platforms such as TikTok and Bilibili have become significant sources of public health information, not only in China but globally.^[9,10]^ However, limited oversight raises concerns about the accuracy and reliability of health-related videos on these platforms.^[11]^ Evaluations of short videos on pancreatic, lung, and liver cancers have reported highly variable quality and reliability.^[12–14]^

Previous studies have evaluated nasopharyngeal cancer-related videos on YouTube, revealing significant variations in video quality.^[15]^ Additionally, an assessment of publicly available English language online information about nasopharyngeal cancer using 3 search engines demonstrated considerable disparities in information quality and poor readability.^[16]^ To date, no studies have evaluated the quality of nasopharyngeal cancer-related videos on TikTok and Bilibili. This study aims to assess the content, quality, and credibility of nasopharyngeal carcinoma-related videos and provide recommendations for improving future health communication strategies.

## 2. Materials and methods

### 2.1. Ethical considerations

The data used in this study were sourced from publicly available video content published on platforms such as Bilibili and TikTok. These videos are publicly accessible, and no personal privacy information was involved during the data collection process. All analyzed content was publicly available, and the study did not involve the collection or processing of users’ private information. In accordance with relevant ethical review guidelines, ethical approval for this study was not required.

### 2.2. Search strategy

In this cross-sectional study, we designed the following search strategy based on previous studies.^[17–19]^ The video search was completed on October 1, 2025, in Yancheng City, Jiangsu Province, China. To minimize the potential impact of algorithmic personalization, browser history, cached files, and cookies were thoroughly cleared before initiating the searches, and all searches were performed while logged out and using newly created accounts. Search results for each platform are sorted according to a default comprehensive ranking.

We searched for relevant videos using the Chinese keyword “nasopharyngeal cancer” on TikTok and Bilibili. We excluded duplicate videos, irrelevant topics, and videos from deactivated accounts until we had collected 100 videos on each platform. Restricting our analysis to the top 100 videos serves 2 purposes. Firstly, videos exceeding this threshold have no significant impact on the analysis.^[20]^ Secondly, the top 100 videos are considered to exert a greater influence on the audience.^[21]^ During the screening process, 4 videos (Irrelevant to the subject) were excluded on TikTok, and 21 videos (Irrelevant to the subject (n = 12), Duplicated videos (n = 8), Account Cancellation (n = 1)) were excluded on Bilibili. Between 1 October, 2025 and 3 October, 2025, we completed video searches, metric extraction, and quality assessments.

### 2.3. data extraction

We recorded detailed information about the selected videos. This information included video source (Bilibili or TikTok), authorship, video duration (in seconds), likes, comments, shares, and collections. Engagement metrics include likes, collections, comments, and shares. We categorized uploaders based on previous research into 4 groups: healthcare professionals, science communicators, general users, and organizational users.^[22,23]^ Specifically, healthcare professionals include physicians and other medical personnel; science communicators include popular science writers and content creators; general users encompass patients or family members; and organizational users represent medical institutions, government agencies, educational institutions, and similar organizations. Firstly, identification was conducted based on verification information displayed on the account homepage. Secondly, for accounts with incomplete or ambiguous information, researchers determined their group by examining the account’s historical video content and comment interactions on the platform. All videos are searched, and metrics are collected by 1 person and completed within 1 day to minimize errors caused by platform algorithm recommendations and video updates.

### 2.4. Video quality assessment

Prior to evaluating the videos, 2 independent researchers reviewed guidelines related to nasopharyngeal cancer, the global quality score (GQS), and the modified DISCERN (mDISCERN) tool. The researchers engaged in discussions regarding these materials to familiarize themselves with and standardize their understanding, thereby minimizing cognitive biases. In this study, the quality and reliability of short videos were assessed using the GQS and the mDISCERN tool. The GQS is a widely used video scoring tool that evaluates the quality of a video through a 5-point Likert scale, with scores ranging from 1 (poor quality) to 5 (high-quality).^[21,24]^ Further details are provided in Table 1. The reliability of the video content was assessed using the mDISCERN tool. It takes the form of 5 questions that are scored based on a “yes” or “no” response. The lowest score is 0, and the highest score is 5, with higher scores indicating higher reliability.^[25]^ Further details are provided in Table 2. Also, we assessed the completeness of the video content. Video content completeness was assessed based on whether the videos covered the following 5 aspects: epidemiology (such as geographical distribution, racial differences, and age distribution); clinical manifestations (such as nasal congestion, bloody nasal discharge, and tinnitus); diagnosis (such as imaging studies, pathological examination, and laboratory indicators); treatment (such as medical therapy, radiotherapy, and surgical intervention); and prognosis (such as survival duration, quality of life, disease progression). A video was considered to cover all 5 domains (epidemiology, clinical manifestations, diagnosis, treatment, and prognosis) if it mentioned age distribution, tinnitus, laboratory indicators, radiotherapy, and survival duration. In contrast, a video that mentioned only geographical distribution and racial differences was considered to address epidemiology only. Given the brevity of the videos and the complexity of medical expertise, our assessment of video content is categorized as either mentioning or not mentioning the subject. If a video touches upon the relevant content in a superficial manner, this study also considers it to cover that topic. The content quality of the videos was recorded and assessed by 2 independent reviewers, and in case of disagreement, a third researcher was co-negotiated to resolve the issue.

**Table 1:** The global quality score quality criteria.

Item features	Points
Poor quality; poor flow of the videos; most information missing; not at all useful for patients	1
Generally poor quality; some information is listed, but many important topics are missing; of very limited use to patients	2
Moderate quality; suboptimal flow; some important topics adequately discussed, but other information poorly discussed; somewhat useful for patients	3
Good quality and generally good flow; most of the relevant information is listed, but some topics are not covered; useful for patients	4
Excellent quality and flow; very useful for patients	5

* (1 to 5 points, representing increasingly higher quality.)

**Table 2:** The modified DISCERN quality criteria.

Reliability score
1. Is the video clear, concise, and understandable?
2. Are valid sources cited?
3. Is the content presented balanced and unbiased?
4. Are additional sources of content listed for patient reference?
5. Are areas of uncertainty mentioned?

* (1 point for answer “yes,” 0 point for answer “no”; The higher the total score, the more reliable it is.)

### 2.5. Statistical analysis

Categorical variables are expressed as frequencies and percentages, while continuous variables are described using the median (interquartile range [IQR]). The chi-square test or Fisher’s exact test was used to assess differences in platform distribution. The Mann–Whitney *U* test was employed for nonparametric comparisons between 2 independent groups, while the Kruskal-Wallis *H* test was used for comparisons among 3 or more groups. Interrater reliability between the 2 independent reviewers was assessed using Cohen’s kappa coefficient, with values interpreted as follows: <0.20, poor; 0.21 to 0.40, fair; 0.41 to 0.60, moderate; 0.61 to 0.80, good; and >0.81, excellent.^[17]^ We used Cohen’s kappa coefficient to assess the level of agreement between reviewers. The kappa coefficient for each evaluation item exceeded 0.7, indicating a high degree of consistency. Spearman correlation analysis was applied to evaluate associations between video-related variables, mDISCERN, and GQS scores across 200 videos.^[26,27]^ In Spearman correlation analysis, the intensity classification of the correlation coefficient (*r*) commonly includes levels such as: 0.00–0.19 (very weak), 0.20–0.39 (weak), 0.40–0.59 (moderate), 0.60–0.79 (strong), and 0.80–1.00 (very strong) correlation.^[28]^ A *P*-value < .05 was considered statistically significant.

## 3. Results

### 3.1. Basic characteristics of video

**Figure 1.**
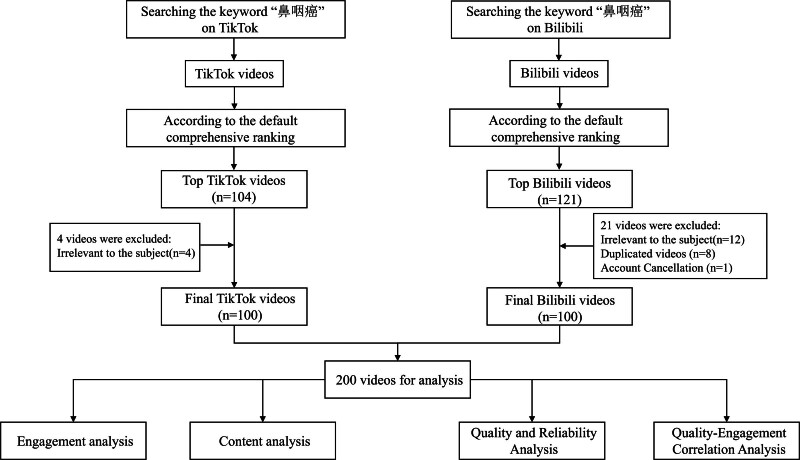
Flowchart of the selection process for nasopharyngeal cancer-related videos.

Based on the established inclusion and exclusion criteria, we ultimately selected 100 videos from each platform for further data extraction and analysis. The detailed selection process is illustrated in Figure 1. Table 3 shows the baseline characteristics of the 200 included nasopharyngeal cancer-related short videos. By uploader category, healthcare professionals accounted for 58.50%, followed by science communicators (19.50%), general users (14.00%), and organizational users (8.00%). The median video duration was 135 s (IQR 68–270). Median engagement metrics were 368 likes (IQR 16–1159), 116 collections (IQR 14–402), 39 comments (IQR 2–233), and 79 shares (IQR 4–320). The median GQS score was 3 (IQR 3–4), and the median mDISCERN score was 2 (IQR 2–2).

**Table 3:** General characteristics, quality, and reliability of the videos.

Variables	Total (n = 200)	Bilibili (n = 100)	TikTok (n = 100)	*P*-value
Video source [n (%)]				<.001
Healthcare professionals	117 (58.5)	36 (36.00)	81 (81.00)	
Organizational users	16 (8.0)	9 (9.00)	7 (7.00)	
General users	28 (14.0)	18 (18.00)	10 (10.00)	
Science communicators	39 (19.5)	37 (37.00)	2 (2.00)	
General information				
Video duration [s, median (IQR)]	135 (68, 270)	165 (81, 589)	121 (55, 210)	.002
Number of likes [median (IQR)]	368 (16, 1159)	16 (3, 123)	1018 (470, 2024)	<.001
Number of collections [median (IQR)]	116 (14, 402)	14 (3, 122)	313 (108, 767)	<.001
Number of comments [median (IQR)]	39 (2, 233)	2 (0, 21)	206 (65, 581)	<.001
Number of shares [median (IQR)]	79 (4, 320)	5 (0, 38)	282 (131, 844)	<.001
GQS scores [median (IQR)]	3 (3, 4)	3 (3, 3)	3 (3, 4)	.093
mDISCERN scores [median (IQR)]	2 (2, 2)	2 (2, 2)	2 (2, 2)	.382

GQS = global quality score, IQR = interquartile range, mDISCERN = modified DISCERN.

### 3.2. Comparison of features across platforms

Platform-specific characteristics are shown in Table 3. Uploader composition differed significantly between TikTok and Bilibili (*P* < .001). Bilibili videos were longer than TikTok videos (*P* = .002). TikTok videos showed higher engagement than Bilibili videos across likes, collections, comments, and shares (all *P* < .001). GQS and mDISCERN scores did not differ significantly between platforms (GQS *P* = .093; mDISCERN *P* = .382).

### 3.3. Video content

**Figure 2.**
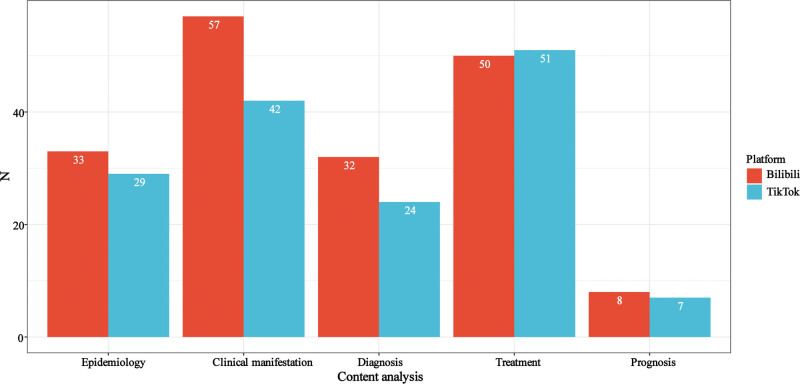
Information on nasopharyngeal cancer-related video content on TikTok and Bilibili.

In the video content analysis section, as shown in Figure 2, videos on the Bilibili platform most frequently covered clinical manifestation, followed by treatment. Videos on the TikTok platform most frequently covered treatment, followed by clinical manifestation. Notably, only a small number of videos on both Bilibili and TikTok mentioned Prognosis.

### 3.4. Engagement metrics based on the type of uploader on both BiliBili and TikTok

Table 4 presents a comparison of video characteristics across different uploaders. Healthcare professionals had significantly shorter videos (105s), followed by general users (169s), organizational users (287s), while science communicators had the longest videos (582s). (*P* < .001) Likewise, Healthcare professionals had a significantly higher number of collections (182), followed by general users (153), organizational users (57), while science communicators had the lowest number of collections (36). (*P* = .02) Similarly, Healthcare professionals had a significantly higher number of shares (146), followed by general users (104), organizational users (85), while science communicators had the lowest number of shares (12). (*P* < .001). Conversely, general users had a significantly higher number of likes (830) when compared to healthcare professionals (522), organizational users (121), and science communicators (15). (*P* < .001) Furthermore, general users had a significantly higher number of comments (236) when compared to healthcare professionals (106), organizational users (6), and science communicators (1). (*P* < .001)

**Table 4:** Engagement metrics based on type of uploader on both BiliBili and TikTok.

Variables	Healthcare professionals (n = 117)	Organizational users (n = 16)	General users (n = 28)	Science communicators (n = 39)	*P*-value
Video duration [s, median (IQR)]	105 (55,187)	287 (104,544)	169 (121,232)	582 (124,1328)	<.001
Number of likes [median (IQR)]	522 (70,1227)	121 (3,1240)	830 (70,1946)	15 (2104)	<.001
Number of collections [median (IQR)]	182 (23,433)	57 (7824)	153 (54,545)	36 (7143)	.02
Number of comments [median (IQR)]	106 (8281)	6 (1,65)	236 (29,894)	1 (0,11)	<.001
Number of shares [median (IQR)]	146 (11,466)	85 (3254)	104 (13,379)	12 (1,45)	<.001

IQR = interquartile range.

### 3.5. Engagement metrics based on the type of uploader on BiliBili

Table 5 presents a comparison of video characteristics across different uploaders on Bilibili. General users had a significantly higher number of likes (242) when compared to science communicators (13), healthcare professionals (6), and organizational users (3). (*P* < .001) Likewise, general users had a significantly higher number of collections (84), followed by science communicators (34), organizational users (8), while healthcare professionals had the lowest number of collections (4). (*P* = .002) Similarly, general users had a significantly higher number of comments (54) when compared to healthcare professionals (1), organizational users (1), and science communicators (1). (*P* < .001) Furthermore, general users had a significantly higher number of shares (14) when compared to science communicators (9), organizational users (4), and healthcare professionals (2). (*P* = .037) Conversely, healthcare professionals had significantly shorter videos (80s), followed by organizational users (153s), general users (182s), while science communicators had the longest videos (610s). (*P* < .001)

**Table 5:** Engagement metrics based on type of uploader on BiliBili.

Variables	Healthcare professionals (n = 36)	Organizational users (n = 9)	General users (n = 18)	Science communicators (n = 37)	*P*-value
Video duration [s, median (IQR)]	80 (62,121)	153 (122,325)	182 (164,317)	610 (134,1398)	<.001
Number of likes [median (IQR)]	6 (2,69)	3 (2,17)	242 (55,1155)	13 (1,90)	<.001
Number of collections [median (IQR)]	4 (1,23)	8 (3,13)	84 (32,168)	34 (5132)	.002
Number of comments [median (IQR)]	1 (0,14)	1 (0,5)	54 (9165)	1 (0,8)	<.001
Number of shares [median (IQR)]	2 (0,22)	4 (0,14)	14 (10,99)	9 (1,38)	.037

IQR = interquartile range.

### 3.6. Engagement metrics based on the type of uploader on TikTok

Table 6 presents a comparison of video characteristics across different uploaders on TikTok. Science communicators had a significantly higher number of likes (10853) when compared to general users (2029), organizational users (1172), and healthcare professionals (917). (*P* = .024) Likewise, science communicators had a significantly higher number of collections (3180), followed by organizational users (777), general users (560), while healthcare professionals had the lowest number of collections (292). (*P* = .031) Similarly, science communicators had a significantly higher number of shares (2170) when compared to general users (491), healthcare professionals (265), and organizational users (248). (*P* = .234) Furthermore, general users had a significantly higher number of comments (1152) when compared to science communicators (435), organizational users (53), and healthcare professionals (178). (*P* < .001) Conversely, healthcare professionals had significantly shorter videos (112s), followed by general users (118s), science communicators (195s), while organizational users had the longest videos (474s). (*P* = .206)

**Table 6:** Engagement metrics based on type of uploader on TikTok.

Variables	Healthcare professionals (n = 81)	Organizational users (n = 7)	General users (n = 10)	Science communicators (n = 2)	*P*-value
Video duration [s, median (IQR)]	112 (52,205)	474 (214,551)	118 (84,168)	195 (154,236)	.206
Number of likes [median (IQR)]	917 (440,1742)	1172 (473,3808)	2029 (1134,2781)	10853 (7503,14202)	.024
Number of collections [median (IQR)]	292 (93,557)	777 (249,1403)	560 (286,734)	3180 (2384,3975)	.031
Number of comments [median (IQR)]	178 (63,345)	53 (27,158)	1152 (710,1423)	435 (326,543)	<.001
Number of shares [median (IQR)]	265 (96,825)	248 (170,709)	491 (299,845)	2170 (1361,2978)	.234

IQR = interquartile range.

### 3.7. Video quality across different uploaders

**Figure 3.**
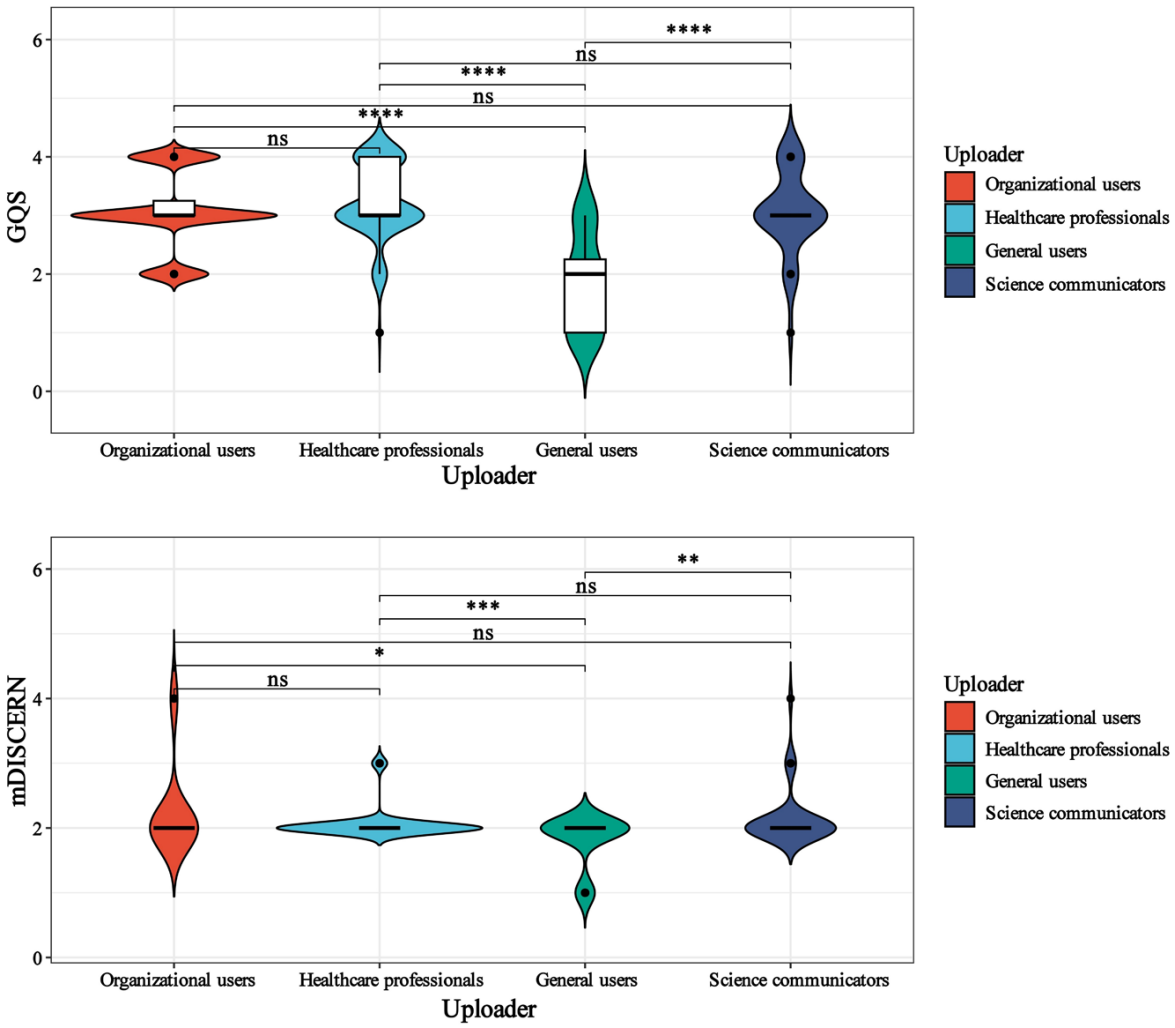
Distribution of quality and reliability scores across different types of uploader groups. * indicates *P* < .05, ** indicates *P* < .01, *** indicates *P* < .001, **** indicates *P* < .0001, ns indicates not significant. GQS = global quality score.

Figure 3 indicates that videos uploaded by healthcare professionals received higher GQS scores. Organizational users and science communicators achieved higher mDISCERN scores. Compared to general users, healthcare professionals’ videos scored higher on both GQS and mDISCERN, while no significant differences were observed between organizational users, science communicators, and healthcare professionals.

### 3.8. Correlation analysis

**Figure 4.**
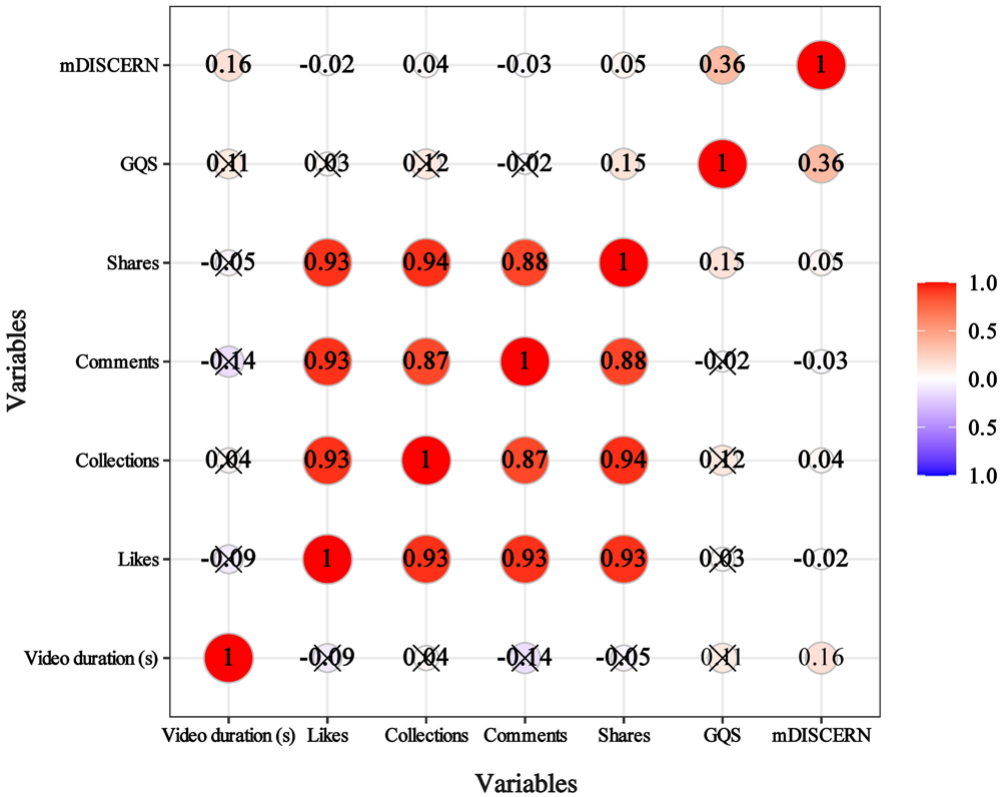
Spearman correlation analysis between different video variables, GQS, and mDISCERN scores. GQS = global quality score.

We employed Spearman correlation analysis to examine the relationship between video interaction metrics (such as likes, comments, saves, and shares) and GQS, as well as mDISCERN scores (Fig. 4). The findings revealed significant positive correlations among interaction metrics (*R* > 0.8, *P* < .05). The GQS showed a positive correlation with the number of video shares (*R* = 0.15, *P* < .05). The mDISCERN scores demonstrated positive correlations with the number of video collections (*R* = 0.04), video shares (*R* = 0.05) and video duration (*R* = 0.16, all *P* < .05).

## 4. Discussion

The aim of this study was to evaluate the content, quality, and reliability of nasopharyngeal cancer-related short videos on 2 major Chinese platforms, TikTok and Bilibili. Based on the analysis of 200 videos, 4 principal findings emerged. First, uploader composition and engagement metrics differed markedly between platforms. Second, the overall quality and reliability of nasopharyngeal cancer-related videos were poor. Third, content coverage was unbalanced, with prognosis rarely addressed. Fourth, user engagement metrics did not reliably reflect objective quality or reliability.

Results showed that on Bilibili, uploaders were primarily healthcare professionals (36%) and science communicators (37%). On TikTok, healthcare professionals were the primary uploaders (81%). This is consistent with previous research findings. TikTok has implemented stricter regulations on video content, stipulating that only accredited institutions and doctors may upload medical content.^[22]^ In this study, engagement metrics such as likes, collections, shares, and comments were higher on TikTok, though video durations were shorter than on Bilibili. This is a phenomenon associated with the platform’s characteristics.^[29]^ Research suggests that TikTok’s videos are notably brief, typically lasting between 15 and 60 seconds, making them conducive to rapid dissemination. In contrast, Bilibili’s longer videos require viewer patience, potentially reducing engagement.^[17]^ The platform should establish a video screening mechanism to prioritize the display of professional, high-quality video content in search results, ensuring the dissemination of accurate knowledge.

We employed the GQS and mDISCERN score to evaluate the quality and reliability of video content across various platforms. The median GQS score for 200 videos was 3 (IQR 3–4), while the median mDISCERN score was 2 (IQR 2–2). Results indicate that videos related to nasopharyngeal carcinoma exhibit lower overall quality. This finding aligns with previous research.^[30]^ This phenomenon is primarily attributed to the inherent characteristics of short videos: their concise and singular style limits the scope and depth of video content.^[31]^ Although the video quality scores across the 2 platforms did not differ significantly, Bilibili’s videos appeared to be of lower quality than those on TikTok. This is consistent with the findings of Zheng et al.^[14]^ This disparity may stem from TikTok’s predominantly healthcare professional uploaders. Research indicates that health videos uploaded by healthcare professionals are characterized by high content quality and credibility.^[32]^

We found that video content on nasopharyngeal carcinoma across both platforms primarily covered clinical manifestations and treatment. However, some videos provided overly simplified symptom descriptions, such as bloody nasal discharge, without detailing comprehensive diagnostic processes. This may lead viewers to misinterpret common physiological changes as nasopharyngeal carcinoma, triggering excessive anxiety and panic.^[19,32]^ Some videos briefly describe treatment options without addressing potential adverse reactions, resulting in a narrow understanding among patients.^[33]^ Notably, over half of the videos fail to mention reports related to nasopharyngeal carcinoma prognosis. One possible explanation is that prognosis involves multiple factors with significant individual variation, requiring comprehensive interpretation based on treatment plans, tumor staging, and laboratory indicators.^[34,35]^ Future educational videos should address this to provide viewers with a more holistic understanding of nasopharyngeal carcinoma and foster greater societal awareness for patients.

We also found that videos uploaded by general users performed better in terms of likes and comments. Previous research shows that general users achieve better engagement metrics by sharing personal experiences of illness that resonate with their audience, offering neutral and relatable insights.^[17]^ Science communicators’ videos have the longest duration, but lengthy videos require viewers to maintain their patience throughout. Healthcare professionals’ videos have more collections and shares. This indicates that the accumulation of expertise is crucial for medical information videos. However, compared to videos by healthcare professionals, those by general users had significantly lower GQS scores and mDISCERN scores. Regarding mDISCERN scores, healthcare professionals’ videos scored lower than those from organizational users and science communicators. This may stem from healthcare professionals prioritizing conciseness while neglecting source citations.^[36,37]^ Healthcare professionals should incorporate more guideline references and recent research findings into their videos to enhance professionalism and credibility.^[22,38]^

Additionally, Spearman correlation analysis revealed a significant positive correlation among likes, comments, collections, and shares, consistent with findings from numerous studies, including those by Guan et al.^[39]^ This indicates synergistic effects in video interaction data, where highly engaged videos typically excel across multiple dimensions.^[40]^ However, the interaction data is poorly or negatively correlated with video quality and reliability.^[41,42]^ This aligns with prior research indicating that user engagement is more influenced by content popularity than by content quality.^[43]^ Users struggle to discern the quality of health science videos through interaction metrics alone.^[17]^ This underscores the necessity of enhancing the dissemination of high-quality medical science videos, as misinformation can generate harmful societal impacts.^[42]^ Consequently, platforms should optimize review mechanisms and recommendation algorithms for medical science videos to increase the reach of reputable content.

The primary significance of this study lies in providing a comprehensive methodology for evaluating video quality on short-form video platforms. This study employed the following measures to enhance the validity and credibility of its findings. Firstly, cross-platform comparisons (TikTok and Bilibili) mitigated platform-specific biases, rendering conclusions more robust across diverse social media environments. Secondly, prespecified retrieval strategies reduced selection bias and improved replicability. Thirdly, the use of established and validated measurement tools (GQS and mDISCERN) ensured greater reliability in assessing video quality and credibility. Fourthly, dual independent rater scoring, supplemented by consistency checks and disagreement arbitration, minimized subjectivity and scoring bias. Finally, correlational analysis between multidimensional interaction metrics and video quality scores bolstered the overall credibility of the findings. By comparing videos from TikTok and Bilibili, we analyzed how platform characteristics, video sources, and interaction data influence the dissemination quality of health information. Furthermore, utilizing the GQS and mDISCERN tools, this research conducted objective evaluations of health information videos, offering evidence for public health policy formulation, regulatory mechanisms for short-form video platforms, and the direction of medical science communication.

Our study has some limitations. First, the videos were collected from Chinese-language platforms, which may limit generalizability to other languages and cultures. Second, platform rankings and engagement metrics can change over time, and a short capture window may affect representativeness. Third, video age may confound engagement comparisons because older videos have had more time to accumulate likes, comments, shares, and collections, whereas newer videos may not reflect long-term audience behavior.^[44,45]^ Fourth, Video content and quality ratings rely on manual assessment, which may introduce subjective bias and affect replicability. Future research should consider incorporating Artificial Intelligence-driven analytical tools to reduce subjective bias.

## 5. Conclusion

This study analyzed the characteristics and quality of 200 nasopharyngeal cancer-related videos from 2 short video platforms (TikTok and Bilibili). The results indicate significant differences in uploader composition and interaction patterns between the 2 platforms, with TikTok exhibiting higher engagement despite shorter videos. Overall video quality and reliability remain at a moderately low level. Content coverage is uneven, with pronounced deficiencies in prognosis. These findings underscore that while short video platforms hold considerable potential for public health education on nasopharyngeal cancer, issues such as improving content quality and optimizing platform recommendation mechanisms require attention.

## Acknowledgements

The authors would like to express their gratitude to the participants who participated in the study.

## Author contributions

**Conceptualization:** Yang Miao, Tingting Shen, Ye Hu.

**Data curation:** Yang Miao, Ye Hu.

**Formal analysis:** Hui Wu, Qineng Gong, Tingting Shen, Ye Hu.

**Methodology:** Qineng Gong, Tingting Shen.

**Investigation:** Tingting Shen.

**Writing – review & editing:** Qineng Gong, Linlin Zhang, Ye Hu.

**Writing – original draft:** Yang Miao, Hui Wu, Qineng Gong, Linlin Zhang, Ye Hu.
